# Genome‐Edited Maize Expressing Two Native Genes Confers Broad‐Spectrum Resistance to Northern Corn Leaf Blight

**DOI:** 10.1111/mpp.70205

**Published:** 2026-02-11

**Authors:** Huirong Gao, Bailin Li, Kevin Fengler, Meizhu Yang, Megan Schroder, Melissa Rahe, Nathalie Sanyour‐Doyel, Julie Qi, Victor LIaca, Mary Beatty, Wang‐Nan Hu, Brittany Barrett, Bret Norman, Hua Mo, April Leonard, Bill Wilson, Robert B. Meeley, Leandro Perugini, Nandini Krishnamurthy, Jeffrey E. Habben, Girma Tabor

**Affiliations:** ^1^ Research and Development Corteva Agriscience Johnston Iowa USA

**Keywords:** allele swap, cisgene, CRISPR, genetic stack, HDR, NCLB

## Abstract

Northern corn leaf blight (NCLB) can result in yield losses of up to 50% in maize. The most effective strategy for managing NCLB is the deployment of resistant hybrids. Conventional breeding methods typically require 6 or 7 backcross generations to introgress a resistance locus, often bringing along undesirable traits that reduce yield. Recent advances in genome editing offer a precise alternative, enabling the targeted incorporation of resistance genes without linkage drag. In this study, we identified an NCLB resistance gene, NLB18‐R, that is allelic to *Htn1* and *Ht2/Ht3*. Using CRISPR‐Cas9, we replaced the susceptible allele (NLB18‐S) with NLB18‐R in an elite inbred, resulting in enhanced resistance to NCLB. In a parallel experiment, we inserted both NLB18‐R and the resistance gene Ht1‐R into preselected, closely linked sites on chromosome 1. Through genetic crossing, we combined these edits into a stack. The resulting genome‐edited plants exhibited resistance to *Setosphaeria turcica* races 0, 1 and 23N. Field trials under disease‐free conditions showed no significant yield differences between hybrids carrying NLB18‐R, Ht1‐R, or the stack compared to null and wild‐type controls. These findings demonstrate that CRISPR‐Cas9‐mediated genome editing is a powerful tool for rapidly developing commercial‐grade maize hybrids with broad‐spectrum resistance to NCLB, and potentially other diseases.

## Graphical abstract

We identified a novel resistance allele, NLB18‐R, at the *Htn1* locus. Using CRISPR‐Cas9, we replaced the susceptible allele (NLB18‐S) with NLB18‐R in an elite inbred. We stacked NLB18‐R and the resistance gene Ht1‐R. The resulting genome‐edited plants exhibited resistance to *Setosphaeria turcica* races 0, 1 and 23N.

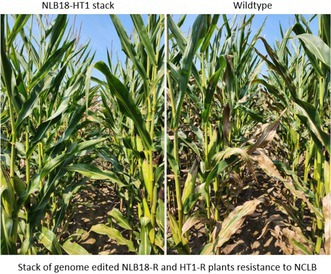

## 1 Introduction

Northern corn leaf blight (NCLB), caused by *Exserohilum turcicum* (teleomorph *Setosphaeria turcica*), is a widespread foliar disease that impacts corn (maize) production globally (Hooker 1963; Leonard et al. 1989; Simcox and Bennetzen 1993; Thakur et al. 1989). Under favourable conditions, NCLB can result in a grain yield loss of up to 50% (Raymundo and Hooker 1981). An integrated approach of crop rotation, fungicide application and use of resistant hybrids has been employed to control and mitigate the damage of NCLB (Kloppers and Tweer 2009; Raymundo et al. 1981). Among these approaches, planting resistant corn hybrids is the most economical and effective method.

In North America, NCLB‐resistance in commercial corn hybrids is primarily derived from the race‐specific *Ht1* gene, a coiled‐coil nucleotide‐binding, leucine‐rich repeat (NLR) gene encoding an intracellular immune receptor (Thatcher et al. 2023; Welz and Geiger 2000). However, due to the extensive use of *Ht1* in commercial hybrids, race 1 of *S. turcica*, which overcomes *Ht1*, has become prevalent in the United States (Leonard et al. 1989; Smith and Kinsey 1980; Turner and Johnson 1980; Weems and Bradley 2018). Therefore, deploying hybrids with additional NCLB resistance genes is needed to effectively manage this damaging disease.

Other quantitative trait loci (QTLs) for NCLB resistance have been reported (Welz and Geiger 2000). One major QTL, known as *Htn1*, has been mapped to the long arm of chromosome 8, with two resistant alleles identified (Hurni et al. 2015; Raymundo et al. 1981; Yang et al. 2021). These alleles encode wall‐associated kinases that likely function as a cell surface immune receptor. In North America, most of the commercial corn hybrids have susceptible alleles at the *Htn1* locus. Disease resistance genes such as those in the *Htn1* locus are often found in non‐elite corn lines or closely related, sexually compatible relatives. Traditionally, corn breeders have introduced native resistance genes from these lines into elite corn inbred lines through backcrossing. This trait introgression process typically requires 6 or 7 generations of backcrossing to obtain recurrent, commercially elite parent lines. However, the backcrossing process often introduces undesirable genes that are linked or unlinked to the resistance genes. As a result, the hybrids generated from such a process often yield less or have other undesirable traits (Brown and Rant 2013; Schouten et al. 2006).

As an alternative to backcross breeding, the disease resistance genes can be isolated and directly introduced into elite corn lines using genetic transformation technology. As no other genes are transferred, linkage drag is avoided. The cisgenic approach, where a cisgene including promoter and terminator from native genomic sequences is inserted into elite lines, is most close to traditional breeding (Holme et al. 2012, 2013; Hou et al. 2014; Mullins et al. 2022; Schouten et al. 2006). The cisgenic resistance gene comes from the breeder's gene pool representing a diverse collection of genetic material, from closely related varieties to exotic germplasm, landraces and close relatives that are genetically crossable with the species. Although this approach can rapidly generate resistance gene inbreds and overcome the linkage drag associated with backcrossing, the untargeted transformation method is limited to particle gun co‐delivery of the cisgene and selection marker gene, and also results in the cisgene being inserted randomly into the genome. Multiple resistance genes or traits are needed for commercial product development, but stacking multiple resistance genes that reside at different parts of the genome is challenging and sometimes not possible (Chen and Ow 2017; Petolino and Kumar 2016).

Recent advancements in CRISPR‐Cas genome editing can enable precise introduction of DNA sequences through homology‐directed recombination (HDR). This method allows for accurate replacement or insertion of cisgene without incorporating any undesired sequences (Baltes et al. 2014; Barone et al. 2020; Gao, Gadlage, et al. 2020; Gao, Mutti, et al. 2020; Hummel et al. 2017; Liu et al. 2024; Peterson et al. 2021; Schreiber et al. 2024; Shi et al. 2017).

Here, we report identification, cloning and validation of NLB18‐R, a resistant gene allelic to *Htn1* and Ht2/Ht3 from the proprietary inbred PH26N. Using CRISPR‐Cas9, we replaced the susceptible allele (NLB18‐S) in an elite inbred with the cisgenic NLB18‐R allele at its native locus, and independently inserted NLB18‐R and HT1‐R into preselected genomic locations. Our results demonstrated that these NCLB resistance genes were expressed at expected levels in elite lines, both at their native loci and at the new insertion sites. The CRISPR‐Cas9‐edited plants exhibited efficacy against respective *S. turcica* races, and the genetic stack assembled by crossing NLB18‐R and HT1‐R variants conferred resistance to multiple races of *S. turcica*. Under field conditions without NCLB disease pressure, we further confirmed that the introduced genes did not negatively impact grain yield.

## 2 Results

### 2.1 Identification and Validation of NCLB Resistance Gene From PH26N

**FIGURE 1:**
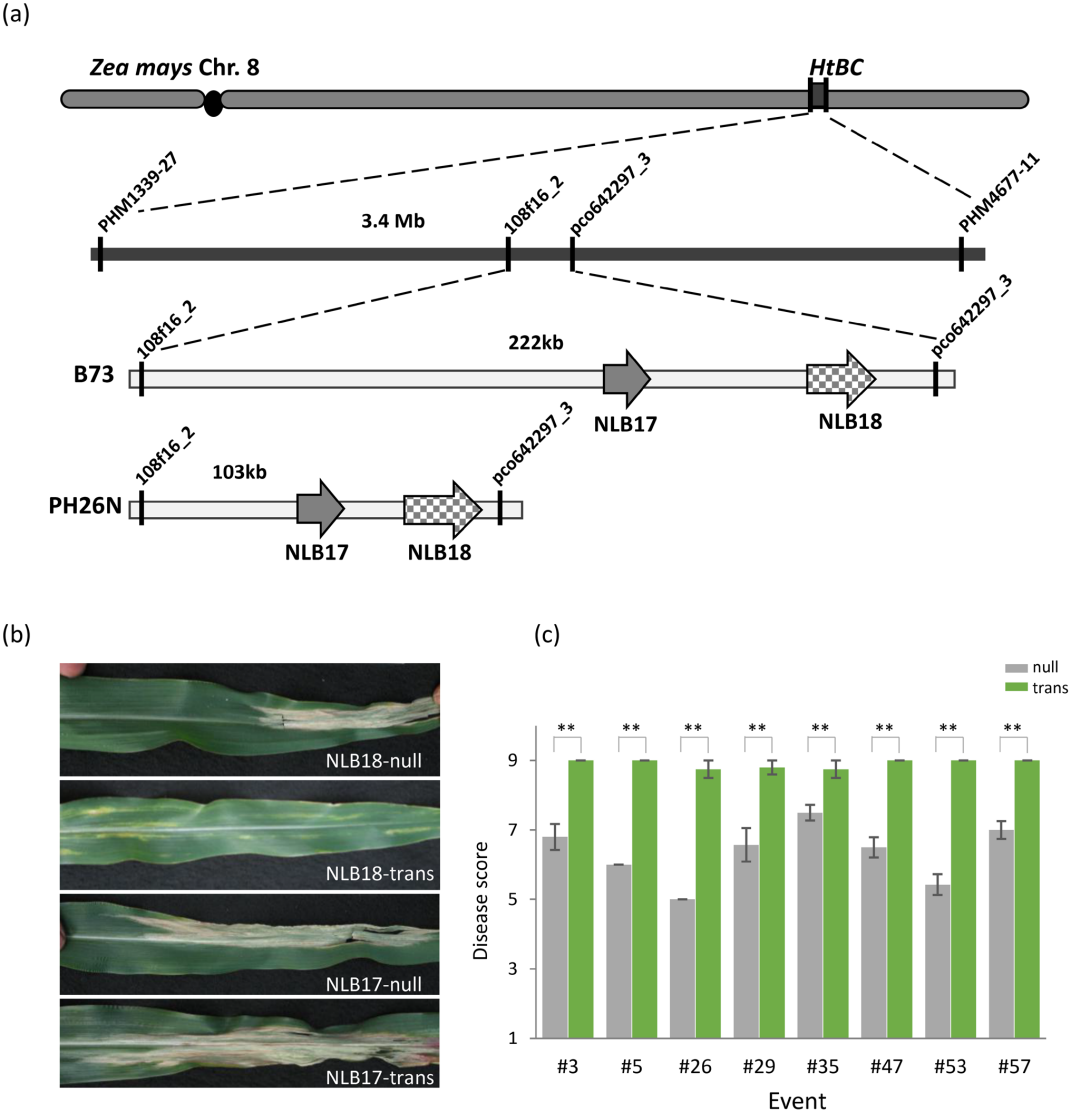
Map‐based cloning and validation of NLB18 with transgenic plants. (a) Schematic representation of the HtBC locus on chromosome 8. The middle panel depicts the region in B73v4 with relevant markers annotated. The bottom panel compares the interval in B73 with that in the resistant donor line PH26N. (b) Leaf response to race 0 of *Setosphaeria turcica* infection. Homozygous bc_0_ (F_2_) plants in HC69 were inoculated at V3–V4 stage and photographed 14 days post‐inoculation in the greenhouse. NLB18‐trans, NLB18‐PH26N CDS; NLB17‐trans, NLB17‐PH26N CDS; null, null segregants. (c) Field evaluation of the NLB18‐PH26N transgenic plants (genomic fragment) against race 0 of *S. turcica*. Trans, homozygous bc_0_ (F_2_) plants in HC69; Null, null segregants. Plants were rated on a 1–9 scale (1 = highly susceptible, 9 = highly resistant). *p* values were derived from a two‐sided Student's *t* test (**p < 0.01). Data are presented as the mean ± SE, *n* = 2–9 plants.

PH26N is a Pioneer inbred line with strong resistance against NCLB. QTL mapping was conducted using a doubled haploid (DH) bc_3_ population of 228 lines constructed with PH26N as the donor and PH581 (NCLB susceptible) as the recurrent parent. A major QTL for NCLB resistance, named HtBC, was detected on chromosome 8, between SNP markers PHM13395‐27 and PHM4677‐11 (Figure 1a, Table S1). Further fine mapping with large BC_4_S_1_ and BC_4_S_2_ populations and additional markers delimited the QTL into a 103 kb interval, between markers 108f16_2 and pco642297_3, in the PH26N genome. Within the interval, there are two annotated wall associated kinase‐like candidate genes, NLB17 and NLB18 (Figure 1a). To determine which of the two genes is responsible for NCLB resistance, transgenic plants were generated to overexpress the coding sequences of NLB17 and NLB18 under control of the moderate constitutive maize H2B promoter. Plants positive for the NLB18 transgene and null segregants were inoculated with race 0 of *S. turcica* in the greenhouse and leaves examined 14 days later. The susceptible and resistant plants, as identified by the presence or absence of typical NCLB necrotic lesions, respectively, perfectly corresponded with the presence or absence of the NLB18 transgene (Figure 1b, Table 1), indicating enhanced resistance in the NLB18 overexpression transgenic plants. However, overexpressing NLB17 did not affect lesion formation in inoculated plants; both the transgenic plants and null segregants showed equivalent symptoms (Figure 1b, Table S2). The NLB18 genomic sequence from PH26N including promoter and terminator was also cloned and randomly introduced into the PHR03 corn inbred. Transgenic events were evaluated in the field for resistance to NCLB. Based on disease severity, inoculated plants were rated on a 1–9 scale with 1 being highly susceptible and 9 highly resistant. For all eight events tested, the NLB18 transgenic plants were resistant to NCLB (Figure 1C). The results of NLB18 overexpression, but not NLB17, enhancing plant resistance to NCLB demonstrated that NLB18 is the causal gene underlying the NCLB resistance in the QTL.

**TABLE 1:** Resistance of NLB18‐R transgenic plants to *Setosphaeria turcica* in the field.

Event	NLB18‐R in plant	No. of resistant plants	No. of susceptible plants
R171122766	Positive	17	0
	Null	0	23
R171122767	Positive	19	0
	Null	0	24
R171122769	Positive	16	0
	Null	0	28
R171122773	Positive	24	0
	Null	0	20
R171135528	Positive	5	0
	Null	0	11

*Note:* Hemizygous NLB18‐R transgenic plants and null segregants were inoculated with race 0 of *S. turcica* and categorised as susceptible or resistant based on presence or absence of characteristic northern corn leaf blight symptoms, respectively.

NLB18 resides at the same genomic location as the previously reported *Htn1* and *Ht2/Ht3*, which are considered to be different resistance alleles of the same QTL (Hurni et al. 2015; Yang et al. 2021). NLB18 from PH26N shares 90.3% and 97.8% amino acid sequence identity with *Htn1* and *Ht2/Ht3*, respectively, representing a new NLB resistance allele for the *Htn1/Ht2/Ht3* locus, from here on referred to as the *Htn* locus. This allele herein is referred to as NLB18‐R. The protein sequence of NLB18‐R is 90.9% identical to the allele in B73, an inbred susceptible to NCLB. However, the B73 allele has a deletion of 14 amino acids in the N‐terminal region (Figure S1). This deletion could interfere with pathogen‐associated molecular pattern (PAMP) recognition, negatively affecting the function of conferring resistance (Nicaise et al. 2009).

### 2.2 Allele Swap to Replace Susceptible NLB18 With a Resistant NLB18 Allele at Its Native Locus Using CRISPR‐Cas9

**FIGURE 2:**
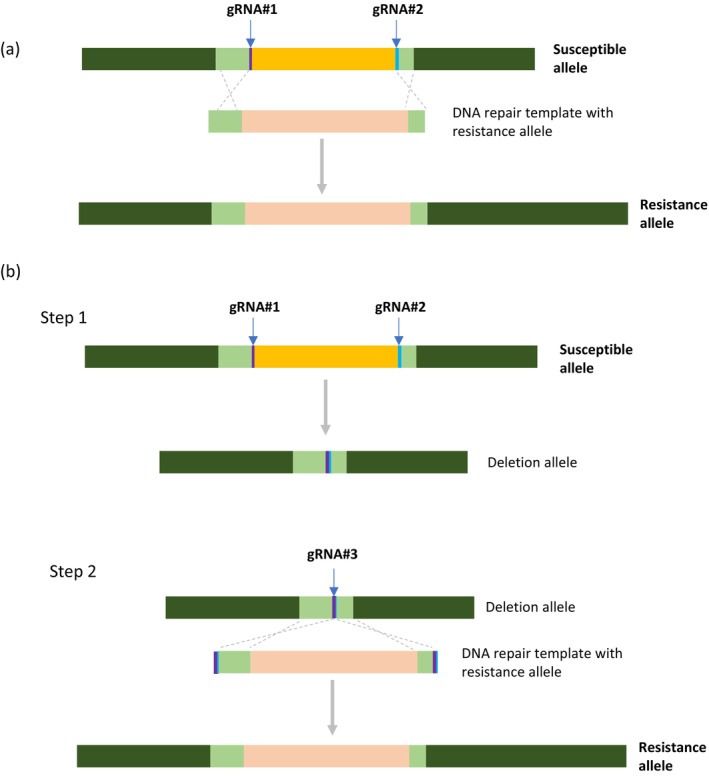
Diagrams of allele swap using CRISPR‐Cas9. (a) Direct replacement of a susceptible allele using a DNA repair template containing a resistance allele. (b) Two‐step replacement strategy with deletion of the susceptible allele first using two gRNAs #1 and #2, followed by insertion of the resistance allele at the newly created junction using gRNA #3 and the DNA repair template.

The NLB18‐R allele was used to develop NCLB resistance hybrids. To avoid linkage drag associated with backcrossing, the CRISPR‐Cas9 technology was employed to replace the susceptible NLB18 allele (NLB18‐S) in elite inbreds with NLB18‐R. Initially, we attempted a direct allele swap by using a construct that contains two gRNAs targeting the genomic sequences that flank the NLB18‐S allele in the recipient corn inbred PH1V5T, and a HDR template consisting of the 13.6 kb genomic sequence of NLB18‐R and the left and right homology arms (Figure 2a). Plasmids encoding gene‐editing reagents of two guide RNAs (gRNA#1 and gRNA#2), *Streptococcus pyogenes* Cas9 and HDR template were introduced into immature embryos using particle bombardment along with plasmids carrying helper genes (Lowe et al. 2016). No plants were recovered with the NLB18‐S allele replaced by NLB18‐R, albeit plants with mutation at TS1 and TS2, or deletion of the NLB18‐S allele were found at high frequencies. This outcome is probably due to the much higher efficiency of nonhomologous end joining (NHEJ) relative to HDR‐facilitated DNA repair in plants (Pacher and Puchta 2017). To achieve an effective allele swap, we then adopted a two‐step strategy: using a plant with deletion of NLB18‐S in the next transformation round for targeted insertion of NLB18‐R (Figure 2b). T_0_ plants containing NLB18‐S deletion with precise repair at the junction (Figure S2) were selected and backcrossed to the wildtype parent PH1V5T to produce bc_0_ (F_1_) progenies. The bc_0_ (F_1_) plants with the NLB18‐S deletion confirmed by sequencing were self‐pollinated and resulting bc_0_ (F_2_) plants were genotyped to identify homozygous plants that also were free from transgenic plasmid DNA in the genome. The homozygous plants, named NLB18‐S‐del, were self‐pollinated again to produce bc_0_ (F_3_). The immature embryos of bc_0_ (F_3_) were used in the second round of transformation for NLB18‐R insertion to complete the allele swap (Figure S3a). In the NLB18‐S‐del plant, a new target site sequence, TS3, formed after NLB18‐S deletion and junction repair via NHEJ (Figures 2b and S2). A guide RNA, gRNA#3 homologous to the newly formed TS3 (Figure 2b), was used to generate a DNA double‐strand break (DSB) for the NLB18‐R insertion. The NLB18‐R allele was supplied by a DNA repair template consisting of the 13.6 kb NLB18‐R sequence flanked by two homology arm sequences (492 bp and 201 bp) that are identical to the DNA sequences upstream and downstream of the TS3 site in the NLB18‐S‐del plant. In the repair template, two TS3 sequences were placed outside of the homology arms to facilitate mobilisation of the DNA repair template (Figure 2b; Peterson et al. 2021). For this round of transformation, the DNA repair template, gRNA#3, Cas9 and the helper genes were assembled into the same T‐DNA and delivered to immature embryos of the NLB18‐S‐del line through *Agrobacterium*‐mediated transformation (Figure S3b).

**FIGURE 3:**
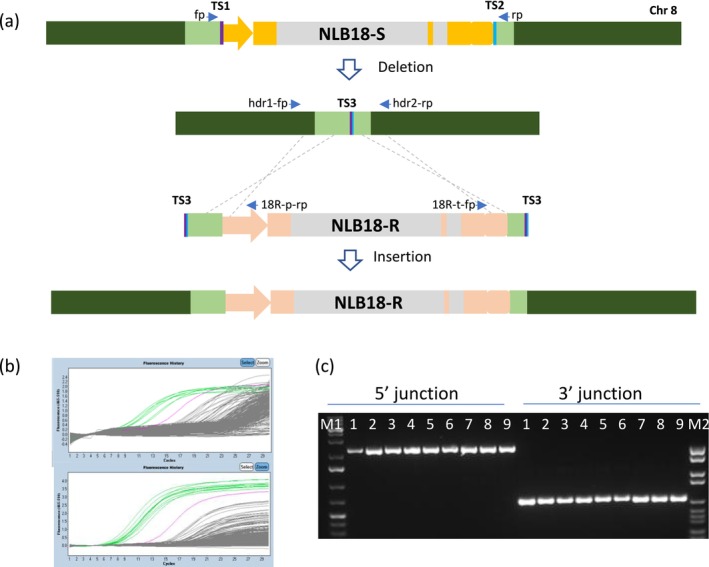
Replacement of the susceptible allele by NLB18‐R at its native locus on chromosome 8. (a) Diagrams showing gRNA target sites and PCR primers to identify NLB18‐S deletion and 2×HDR insertion plants in the 2‐step allele swap. fp/rp, primer pair amplifies deletion junction; hdr1‐fp/18R‐p‐rp and 18R‐t‐fp/hdr2‐rp, primer pairs to amplify 5′ and 3′ end swap junctions respectively. (b) Examples of T_0_ plant screening for insertion events with quantitative PCR (qPCR). Green lines represent positive plants. The upper and lower panels show 5′ and 3′ junction qPCR, respectively. (c) Image of agarose gel showing the junction PCR products from nine plants positive with qPCR. Lane M1 and M2, molecular‐weight size markers.

T_0_ plants were analysed by junction quantitative PCR (qPCR)/PCR and Sanger sequencing to identify plants with targeted insertion of NLB18‐R (Figure 3). Multiple plants positive for both 5′ and 3′ junctions were identified and referred to as 2×HDR (Figure 3b,c). We conducted the *Agrobacterium*‐mediated transformation experiment twice, and similar 2×HDR frequencies were observed (Table 2). We also achieved direct allele swap of NLB18‐S with NLB18‐R in PH1V5T (Table 2) through *Agrobacterium*‐mediated transformation using improved editing designs including replacement of PinII with the OS‐UBI terminator for the Cas9 cassette, increasing the gRNA#1 length to 21 nt, extension of the 5′ homology arm length to 684 bp, and flanking the repair template by the TS2 sequences.

**TABLE 2:** Frequencies of allele swap at the NLB18 locus on chromosome 8 and targeted insertion at preselected sites on chromosome 1.

Experiment	gRNA	Site location	Genotype	Allele to insert	No. of T_0_	No. of 2×HDR T_0_	2×HDR frequency (%)
2‐step allele swap, expt. 1	gRNA#3	Chr08	PH1V5T‐NLB18‐S del	NLB18‐PH26N	1554	11	0.7
2‐step allele swap, expt. 2	gRNA#3	Chr08	PH1V5T‐NLB18‐S del	NLB18‐PH26N	900	10	1.1
Direct allele swap	gRNA#1.1 + 2	Chr08	PH1V5T	NLB18‐PH26N	1530	22	1.4
Targeted cisgenic insertion	gRNA‐45	Chr01, 53.66 cM	PH184C	NLB18‐PH26N	312	5	1.6
Targeted cisgenic insertion	gRNA‐10	Chr01, 54.56 cM	PH184C	HT1‐ED4GP	212	4	1.9

### 2.3 Targeted Insertion of NCLB‐Resistance Genes at Preselected Genomic Sites Using CRISPR‐Cas9

For a robust corn commercial product, it is desirable to use several resistance genes to combat multiple races of a pathogen or multiple plant diseases. Also, co‐locating genes at the same genetic locus would greatly facilitate product assembly. To simplify trait stacking in product development, the concept of complex trait loci (CTLs) has been introduced to handle transgenic traits using the FLP/FRT recombination approach with the pre‐integrated FRT sites (Gao, Mutti, et al. 2020). This strategy can also be applied to native (cisgenic) traits using direct CRISPR‐Cas9‐enabled cisgene integration. Using the CRISPR‐Cas9 technology, individual disease resistance genes can be targeted separately or stacked to preselected sites within a small region on a chromosome to form a CTL. The target sites TS45 and TS10 on chromosome 1 at 53.66 cM and 54.56 cM (predicted position in inbred B73) have previously been successfully used for stacking trait genes (Gao, Mutti, et al. 2020). In this study, they were selected to harbour NLB18‐R and HT1‐R, an *Ht1* resistance allele from Pioneer inbred PH4GP.

For targeted insertion, NLB18‐R and HT1‐R gRNAs and DNA repair templates were designed in a way like that in the NLB18‐R allele swap, as described above (Figure 2b Step 2, Figure S4). The DNA repair template and CRISPR reagents were delivered to immature embryos of PH184C via particle bombardment. Multiple 2×HDR plants were identified in T_0_ for NLB18‐R and HT1‐R insertion. The 2×HDR frequencies are presented in Table 2.

Like the allele swap at the native locus, the genome of T_0_ plants with the cisgene insertion at a preselected site can also contain a selectable marker, Cas9, guide RNA, or helper genes unintended for insertion, yet randomly integrated at different genomic locations. To segregate out the unintended plasmid DNA sequences, the 2×HDR plants were backcrossed twice to the wild type to generate bc_0_ (F_1_) and bc_1_ (F_1_) (Figure S3). Junction qPCR/PCR was used to confirm targeted insertion in bc_0_ (F_1_) (Figure 3), while qPCRs were performed to determine the presence or absence of key unintended elements of the plasmid DNA or T‐DNA. Plants that passed the qPCR analysis were further examined for the absence of unintended plasmid DNA sequences using Southern‐by‐sequencing (SbS) (Zastrow‐Hayes et al. 2015). With this analysis, we found that five of the 2×HDR lines obtained from the 2‐step allele swap experiment contained perfect replacement of the NLB18‐S allele by NLB18‐R and were free from any unintended DNA sequences on the T‐DNA in the genome (Figure S5). Similarly, clean plants with NLB18‐R inserted at TS45 and HT1‐R at TS10 were identified. Selected bc_1_ plants then were self‐pollinated for two generations to produce homozygous and null‐segregant seeds for efficacy testing in greenhouse and field evaluation (Figure S3c).

### 2.4 Stacking NCLB Disease Resistance Cisgenes via Genetic Crossing

To achieve effective control to NCLB, hybrids carrying both NLB18‐R and HT1‐R cisgenes are desirable. To combine these genes, homozygous plants containing NLB18‐R at TS45 and HT1‐R at TS10 were crossed, and the resulting F_1_ plants backcrossed to the wild type PH184C to produce bc_0_ (F_1_) seeds. A total of 3068 bc_0_ (F_1_) seeds were sampled and genotyped by qPCR to determine the presence or absence of NLB18‐R and HT1‐R (Gao, Mutti, et al. 2020). Based on the physical distance of 44 kb between TS45 and TS10 on chromosome 1 in PH184C, most bc_0_ (F_1_) seeds were expected to carry either NLB18‐R or HT1‐R. However, chromosomal crossover between these sites occasionally occurs, resulting in recombinants carrying both cisgenes or neither. Of the 3068 seeds analysed, PCR genotyping revealed that 10 seeds were positive for both cisgenes while 6 were negative, corresponding to a recombination frequency of 0.52%. The frequency was consistent with the physical mapping distance between the two sites and similar recombination rates previously observed when different genes were inserted at these sites (Gao, Mutti, et al. 2020). Seeds positive for both genes were advanced through self‐pollination to produce segregating F_2_ progeny. Homozygous F_3_ seeds and corresponding null segregants derived from these F_2_ plants were subsequently used for efficacy testing.

### 2.5 NLB18‐R and HT1‐R Gene Expression in North American Elite Maize Lines

**FIGURE 4:**
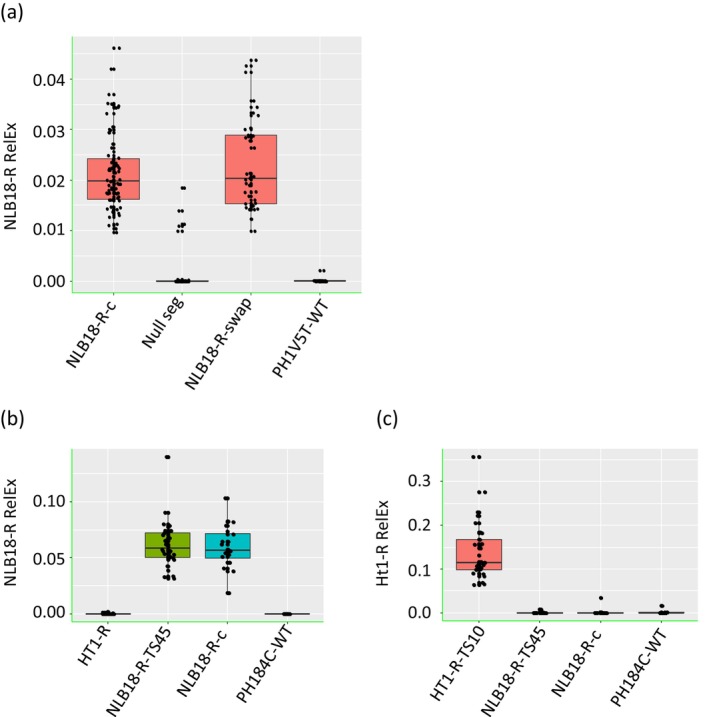
NLB18‐R and HT1‐R genes expressed at native chromosome 8 and chromosome 01 loci in elite maize lines. mRNA expressions of NLB18‐R and HT1‐R in leaves of homozygous BC_0_ (F_2_) plants were measured by reverse transcription‐quantitative PCR. (a) V4 growth stage NLB18‐R swap plants in greenhouse. NLB18‐R‐C, conversion; Null, null segregant of NLB18‐R‐swap. (b and c) Gene expression at V5–V6 growth stage in field. (b) NLB18‐R inserted at TS45. (c) HT1‐R inserted at TS10. Each data point represents a plant. One‐way analysis of variance with Tukey's multiple comparisons test (*p* < 0.05). Eukaryotic translation initiation factor 4G (*eIF4G*) was used as the internal reference.

To assess if the introduced cisgenes were expressed and could confer resistance to NCLB, mRNA expressions of NLB18‐R and HT1‐R in leaves of homozygous bc_0_ (F_2_) plants were measured by reverse transcription‐qPCR (RT‐qPCR). The NLB18‐R transcripts were readily detected in the allele swap lines. The expression levels were comparable among four independent NLB18‐R lines, and there was no significant difference between the allele swap lines and a NLB18‐R line that was generated through the conventional breeding (Figure 4a). NLB18‐S shares significant sequence similarity with NLB18‐R; thus, a weak signal also was detected occasionally in the NLB18‐S segregants and wild‐type plants (Figure 4a). The expression level of the NLB18‐R gene inserted at TS45 in PH184C was similar to that in the conversion line despite being located on a different chromosome (Figure 4b). For the HT1‐R gene inserted at TS10, the transcripts were detected only in the HT1‐R line, as expected (Figure 4c).

### 2.6 NLB18‐R and HT1‐R Conferred Resistance to *S. turcica* Infection

**FIGURE 5:**
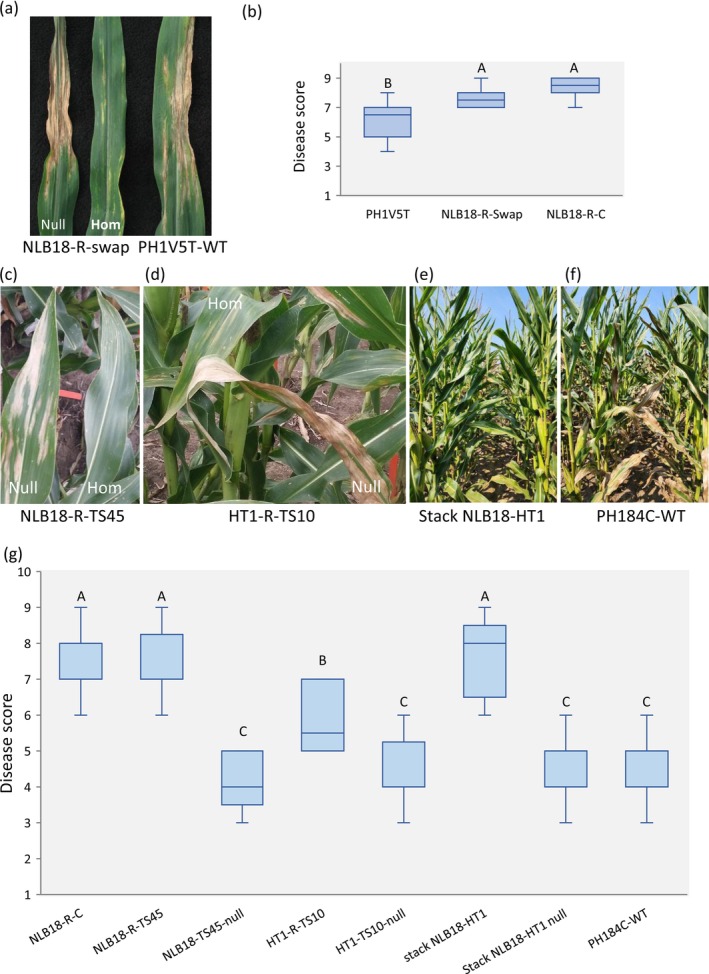
Genome‐edited maize plants carrying NLB18‐R and HT1‐R exhibit resistance to northern corn leaf blight. (a) Greenhouse evaluation of NLB18‐R allele swap (swap) line. BC_0_ (F_2_) plants and PH1V5T wild‐type (WT) control inoculated with race 0 of *Setosphaeria turcica* at the V3–V4 stage and photographed at 14 days post‐inoculation. Hom, homozygous; null, null segregant. (b) Field disease score of NLB18‐R swap lines and controls (PH1V5T and NLB18‐R conversion, NLB18‐R‐C) inoculated with race 0. Plants were rated on a 1–9 scale (1 = highly susceptible, 9 = highly resistant). *n* = 8–22. Different letters denote significant difference (*p* < 0.05, Tukey's HSD). (c and d) Field images of gene‐edited BC_0_ (F_2_) plants inoculated with race 0: (c) NLB18‐R inserted at TS45, (d) HT1‐R inserted at TS10. (e–g) Field evaluation of homozygous BC_0_ (F_3_) plants and controls inoculated with a mixture of race 0 and 1: (e) NLB18‐R and HT1‐R stacks; (f) PH184C WT control. (g) Disease scores for NLB18‐R, HT1‐R and their stack. *n* = 7–22 rows (each data point represents the average score per row). Plants were rated on 1–9 scale. Different letters indicate significant differences (*p* < 0.05, Tukey's HSD). NLB18‐R‐C, conversion line; Null, null segregant; WT, wild type.

Disease severity in corn is influenced by inbred line tolerance to the NCLB pathogen, the prevailing pathogen population and seasonal conditions. To evaluate resistance to NCLB, the bc_0_ (F_2_) plants were inoculated with race 0 of *S. turcica* in greenhouse trials. Homozygous plants carrying the NLB18‐R allele swap did not develop necrotic lesions on leaves 14 days after inoculation, whereas plants with the NLB18‐S allele and the wild‐type inbred PH1V5T displayed typical NCLB symptoms (Figure 5a). In field trials, plants were inoculated with race 0 at the V5–V6 growth stage, and disease severity was assessed later in the season when symptoms typically developed. NLB18‐R plants scored significantly higher for resistance compared with the wild‐type control (*p* < 0.01; Figure 5b). Furthermore, the resistance level of the swap variant did not differ significantly from that of the NLB18‐R conversion line (*p* = 0.445; Figure 5b). Similarly, homozygous plants with NLB18‐R inserted at TS45 or HT1‐R at TS10 exhibited resistance to race 0 (Figure 5c,d).

In a field experiment using a mixture of races 0 and 1 as inoculants, the NLB18‐R insertion variant and the conversion line exhibited high resistance, while the plants carrying the HT1‐R insertion displayed moderate resistance (Figure 5g). The NLB18‐R and HT1‐R stack was highly resistant, whereas null segregants and wild‐type controls were susceptible (Figure 5e–g). In a controlled environment study, plants with NLB18‐R, HT1‐R and their stacks were tested separately against races 0, 1 and 23N. NLB18‐R plants were resistant to races 0 and 1 but susceptible to race 23N, whereas HT1‐R plants were resistant to races 0 and N23 but susceptible to race 1. Notably, the NLB18‐R and HT1‐R stacks conferred resistance to all three races (Table 3). Wild‐type controls were susceptible to all the tested races of *S. turcica*.

**TABLE 3:** NL18‐R and HT1‐R stacks resistant to three races of *Setosphaeria turcica*.

GeName	NLB gene	*S. turcica* race		
		Race 0	Race 1	Race 23N
PH184C	Wild type	S	S	S
DG99507998	HT1_PH4GP	R	S	R
DG99508010	NLB18_PH26N	R	R	S
DM124128026	NLB18_PH26N + HT1_PH4GP	R	R	R

*Note:* Homozygous plants with NLB18‐R at TS45, HT1‐R at TS10, or both resistance genes stacked together via crossing were inoculated with race 0, 1 and 23N in the greenhouse. Plants were rated 14 days after inoculation. S, susceptible; R, resistant, based on presence or absence, respectively, of characteristic northern corn leaf blight symptoms.

### 2.7 Effect of NLB18‐R and HT1‐R Cisgenes on Grain Yield Production Under Non‐NCLB Conditions

The introduction of plant defence genes is commonly assumed to reduce yield in the absence of diseases (Derbyshire et al. 2024), likely due to the metabolic costs of defence mechanisms. Empirical evidence remains limited (Tian et al. 2003; Santa‐Cruz et al. 2014). Much of this assumption originates from breeders' experience introducing resistance genes from non‐elite lines into elite lines via backcrossing. In such cases, it is difficult to determine whether the yield effects are attributable to the resistance gene itself or to linked genomic regions.

**FIGURE 6:**
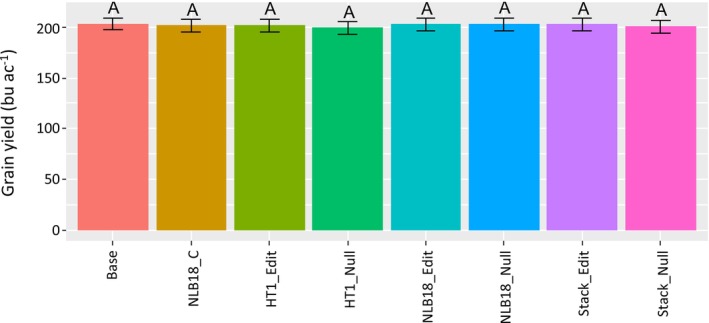
Yield of genome‐edited maize hybrids under conditions without northen corn leaf blight (NCLB) disease pressure. Genome‐edited hybrids made with three female testers were tested across 21 US locations over 3 years. The experiments were monitored for NCLB. Locations where NCLB was detected before kernel physiological maturity were excluded from analysis. Data analyses were conducted using ASREML (VSN International Ltd.) and the values are presented as best linear unbiased estimates (BLUEs). Differences in BLUE across and within nests were calculated, and the *p* value for each contrast was calculated. The same letter, A, on the top of bars indicates no significant difference among tested hybrids (*p* > 0.05). Base, PH184C wild type crossed with testers; NLB18‐C, NLB18‐R conversion. HT1_Edit, HT1‐R inserted at TS10, NLB18_Edite, NLB18‐R inserted at TS45, stack _Edit, NLB18‐R and HT1‐R stacked by crossing, Null, null segregant of each edited hybrid.

Targeted insertion of native resistance genes using CRISPR‐Cas9 genome editing enables evaluation of their impact on yield in the absence of disease pressure. We developed genome‐edited lines carrying NLB18‐R and HT1‐R, as well as a genetic stack of both alleles, and used these to produce hybrid seeds with three testers. These hybrids were assessed for agronomic performance in multilocation and multiyear yield trials conducted under conditions without NCLB. Analysis of yield data from 21 locations over three years showed that hybrids containing NLB18‐R, HT1‐R, or their stacks yielded (*p* > 0.05) similarly to their corresponding null segregants and wild type (*p* > 0.05; Figure 6). Grain moisture of hybrids was also collected. The moisture range was about 19%–20% among the tested hybrids. NLB18‐R conversion hybrid's grain moisture was significantly lower than baseline grain moisture. The genome‐edited and corresponding null segregant showed intermediate moisture levels, mostly overlapping (Figure S6). The reduced grain moisture in the conversion line might result from linkage drag associated with the early‐maturing donor line used during conversion.

## 3 Discussion

In this study, we fine mapped a QTL for NCLB resistance from the proprietary maize inbred PH26N and identified two WAKs as candidate genes. The resistance QTL and candidate genes were disclosed in a patent application filed in 2011 (WO2011163590A1). Subsequently, NLB18‐R was validated as the causal resistance gene, and the transgenic validation result is presented (Figure 1b,c; Table 3). NLB18‐R is a different allele of the subsequently reported *Htn1* and Ht2/Ht3 (Hurni et al. 2015; Yang et al. 2021), based on the genomic location and sequence homology. Moreover, we have not yet identified any pathogen races that can differentiate the three alleles (Htn1, Ht2/Ht3 and NLB18‐R). This resistance allele was used to replace the susceptible one in an elite corn inbred, rendering the elite inbred resistant to NCLB in the greenhouse and under field conditions. Swapping the 13.6‐kb long genomic sequence of NLB18‐R at the native locus is technically challenging, even with the CRISPR‐Cas9 technology. We achieved precise allele replacement with a 2‐step approach: deletion in the first plant transformation, followed by targeted insertion at the newly created junction in the second transformation. We have recently optimised our genome‐editing protocol vector design, allowing us to directly replace the susceptible allele with NLB18‐R at a high frequency in just one transformation. The allele swap technique enables rapid development of cisgenic disease resistance inbreds using native alleles from the breeder's gene pool. The underlying mechanism for allele swap is HDR, which occurs when two molecules of DNA exchange genetic material with each other. This process is identical to what happens in backcross breeding. However, the CRISPR‐Cas9‐mediated breeding method allows for the precise transfer of only the intended DNA sequence, eliminating the linkage drag associated with the conventional trait introgression.

To accelerate hybrid product development, the NCLB resistance genes NLB18‐R and HT1‐R were inserted into preselected sites on chromosome 1 using CRISPR‐Cas9. Due to the proximity of the two sites, genetic crossing was performed to link the two resistance genes through recombination. This allows the genes to be inherited as a single block. Although these resistance genes were not at their native loci, they were expressed at a similar level as at their native genomic location and conferred resistance to NCLB in the elite corn inbred line PH184C. The plants with the two stacked cisgenes through genetic cross showed resistance against three races of *S. turcica* in greenhouse tests. These results highlight the value of CRISPR‐Cas9‐enabled cisgene integration to develop multigenic resistance against one or several diseases. Incorporation of multiple loci to generate lines resistant to multiple races of a pathogen or other pathogens is a daunting task for plant breeders. It not only takes a lot of time and resources, but the chance of producing a commercially relevant line that has resistance to multiple pathogens is very low. The potential linkage drag associated with incorporating multiple loci is prohibitive to developing commercial lines using conventional breeding. The approach we reported here can significantly reduce the time needed to develop lines with multiple disease resistance that are commercially desirable.

Previously, targeted gene insertion and genomic sequence replacement via CRISPR‐Cas9 genome editing were successful in maize with a short piece of DNA of few kilobase pairs in length (Danilo et al. 2018; Gao, Mutti, et al. 2020; Hummel et al. 2017; Shi et al. 2017). An allele replacement is significantly influenced by two factors: the size of the genomic sequence and the chromatin configuration at the location, which determines the accessibility for recombination. However, optimising the CRISPR‐Cas protocol designs can improve the swapping frequency. An efficient generation of DSB is a key factor; we achieved better results by increasing the expression of Cas9 using the maize UBI terminator and when a more effective gRNA with 21 nucleotides was used. Including a mechanism to release the repair template from T‐DNA has been demonstrated to improve HDR template integration (Peterson et al. 2021). In our improved designs, a gRNA target site sequence was placed outside of the homology arms. In addition, a longer homology arm1 was found more effective in allele swapping.

Transferring the complete genomic sequence of a resistance gene, along with its promoter and terminator regions, into its native locus or a well‐characterised, preselected site in the genome can greatly minimise the risk of changing its expression levels, tissue‐specific expression and environmental response. Yield parity, in the absence of NCLB disease demonstrated among lines carrying NLB18‐R, HT1‐R, or combinations of the two genes, with corresponding nulls and wild‐type controls, suggests that the CRISPR‐Cas‐mediated insertion of cisgenes has a significant advantage over conventional breeding for incorporating resistance genes into commercially related inbred lines.

## 4 Experimental Procedures

### 4.1 Identification and Transgenic Validation of NLB18

A DH bc_3_ population of 228 lines was created, with PH26N (NCLB resistant) as the donor and PH581 (NCLB susceptible) as the recurrent parent to map the NCLB resistance QTL. One DH line exhibiting NCLB resistance was selected to backcross again to PH581 and then self‐pollinated. The resulting BC_4_S_1_ population of 730 lines was genotyped and phenotyped for NCLB resistance for the initial QTL mapping. Large BC_4_S_1_ and BC_4_S_2_ populations (total of about 5000 individuals) were generated for further fine mapping. CAPS markers within the QTL interval were developed (Leonard et al. 2014) and used for fine mapping. A PH26N BAC library was constructed, screened and sequenced as previously described (Leonard et al. 2014; Thatcher et al. 2023). Two overlapping BAC clones were identified when the library was screened with the closest flanking markers. Generation of transgenic plants and greenhouse NCLB assay were described (Thatcher et al. 2023).

### 4.2 CRISPR‐Cas9 Target Sites Selection and Plasmid Construction and Maize Transformation

The extended DNA sequence of NLB18 gene in PH1V5T was scanned using proprietary bioinformatic tools. Target sites for *S. pyogenes* Cas9 at the promoter and 3′ UTR of the NLB18 gene were identified as described in Svitashev et al. (2015), target site locations and guide sequences are shown in Figure 3a and Figure S2. TS45 and TS10 on CTL1 selection and sequences were described in Gao, Mutti, et al. (2020) and Figure S4.

The single guide RNA gene consists of a maize U6 polymerase III promoter, a CRISPR RNA, a trans‐activating CRISPR RNA and a terminator. The Cas9 expression cassette contains the maize UBIQUITIN1 promoter (UBI PRO), *S. pyogenes* Cas9 endonuclease and potato protease inhibitor II terminator (PINII). The Cas9 DNA sequence was maize codon‐optimised and the potato ST‐LS1 intron and the nuclear localisation signals from SV40 were added for appropriate expression and nuclear targeting in maize, as previously described (Gao, Gadlage, et al. 2020; Gao, Mutti, et al. 2020; Svitashev et al. 2015). Constructs were assembled using chemically synthesised DNA fragments with standard DNA techniques. Neomycin phosphotransferase II (*NPTII*) served as a transformation selection marker. To improve regeneration of plants, morphogenic regulators *Baby boom* (*Bbm*, also known as ovule development protein2 or *ODP2*) and *Wuschel2* (*Wus2*) were expressed under the control of the maize UBI1 promoter and In2‐2 promoter (Svitashev et al. 2015) or PLTP promoter and Axig1 promoter (Peterson et al. 2021). The CRISPR components and transformation selectable marker were cloned into *Agrobacterium tumefaciens* LBA4404 as described in Peterson et al. (2021). CRISPR component differences among the three allele swap approaches are listed in Figure S7.

*Agrobacterium*‐mediated transformation of maize immature embryos was performed as earlier described (Lowe et al. 2016; Jones et al. 2019; Peterson et al. 2021). To insert or replace NCLB resistant genes to target sites using particle bombardment, biolistic‐mediated transformation of maize immature embryos was performed as previously described (Gao, Gadlage, et al. 2020, Gao, Mutti, et al. 2020; Svitashev et al. 2015).

### 4.3 DNA Extraction and Genotyping by PCR

Genomic DNA was extracted from leaves as described previously (Shi et al. 2017). PCR and next‐generation sequencing were used to detect NLB18‐S allele deletion variants and to sequence the deletion junction. Junction PCR/qPCR assays were used to detect NLB18 resistant gene insertion or replacement at each target site. In this assay, PCR was performed using 2× Extract‐N‐amp PCR Ready Mix (Sigma) or 2× Phusion Flash High‐fidelity PCR Master Mix (Thermo Fisher Scientific), and the nested qPCR was performed using SYBR master mix (Thermo Fisher). The primers are listed in Table S3.

### 4.4 Detection of Plasmid DNA in Plants

The presence of helper genes in the genome of T_1_ plants was determined by qPCR followed by SbS. qPCR was performed using QuantiTect Multiplex PCR Master Mix (Qiagen) with primers and probes listed in Table S4. SbS was performed as described by Zastrow‐Hayes et al. (2015). Illumina whole‐genome sequencing libraries were constructed from DNA derived from plants. Hybridisations and sequencing were carried out as described previously (Zastrow‐Hayes et al. 2015).

### 4.5 RT qPCR to Detect Resistance Gene Expression

Plants were grown in fields in a randomised complete block design. Leaf samples for the NLB18‐R allele swap lines, NLB18‐R and HT1‐R insertion lines and controls were taken from V3–V4 plants. Total RNA was extracted using Omega Bio‐Tek RNA extraction kit, cDNA synthesis was performed using Applied Biosystems High‐Capacity cDNA Reverse Transcription Kit (Thermo Fisher). Real‐time qPCR was performed using Applied Biosystems PowerUp SYBR Green Master Mix (Thermo Fisher) and QuantStudio 7 Flex (Thermo Fisher). The endogenous gene *eIF4G* was used as a reference gene for the assays. Δ*C*_t_ was used to calculate the expression difference between testing genes and the endogenous reference. Primers sequences are listed in Table S5.

### 4.6 Inoculation and Disease Scoring

To assess NCLB resistance, plants were grown either in the greenhouse or in the field. The isolate of *S. turcica* was collected from Iowa and race specificity was confirmed by inoculation differentials. In the greenhouse, plants were inoculated at the V3 stage by applying 200 μL of suspension containing ~10,000 *S. turcica* spores per millilitre. The inoculation method applied to race 0, 1 or 23N. The inoculated plants were then kept in the greenhouse for 2 weeks and monitored for the development of typical NCLB lesions. Based on the presence or absence of the typical lesions, plants were categorised as susceptible or resistant.

In the field, plants were inoculated at the V5–V6 growth stage. Depending on the experiment, plants were inoculated with either race 0 or an equal mixture of race 0 and race 1 by placing a few *S. turcica*‐coated sorghum kernels into the whorls of each plant. After allowing time for the disease to develop throughout the season, the plants were visually scored on a 1–9 scale, ranging from very susceptible (1) to nearly immune (9).

### 4.7 Agronomic Performance of Edited Lines

Hybrids were produced for the eight lines of NLB18‐R at TS45, HT1‐R at TS10, stack of NLB18‐R at TS45 and HT1‐R at TS10, the corresponding null segregants, NLB18‐R conversion line and the wild type of PH184C. Each of the eight lines was crossed with three testers: PH1V69, PHH5G and PH251. The 24 hybrids were used in a multiyear and multilocation yield trial.

Hybrid yield trials were conducted in the United States using a randomised complete design nested by the female tester. Each trial had two replications, with each plot consisting of four adjacent rows; only the middle two rows were harvested for yield assessment. The experiments were carried out at nine locations in 2019, 12 locations in 2020 and 12 locations in 2021.

Experiments were monitored for the presence of NCLB at any growth stage of the plants. Only data from locations without any NCLB incidence were included in the analysis. Additionally, experiments with wind‐ or herbivore‐damaged plots were excluded from the final data collection. Consequently, data from only seven locations in 2019, five in 2020 and nine in 2021 were used for analysis. The number of locations included from the 2020 trials was particularly low due to extensive wind damage at most sites. Data from the following locations were included: Champaign, IL; Garden City, KS; Tifton, GA; Union City, TN; Windfall, IN; York, NE; Woodland, CA; Plainview, TX; Lubbock, TX; and Johnston, IA. In some locations, the experiment was conducted continuously over two or three years.

Analyses were conducted using ASREML (VSN International Ltd.) and the values are presented as BLUEs (Cullis et al. 1998; Gilmour et al. 1995; Gilmour et al. 2009). An observation with a standardised residual that is larger than 4 (in absolute value) is deemed as an outlier. Outlier data points were first detected and removed from the dataset. Four abs(std) were used as a criterion for outlier detection. BLUEs were calculated using ASREML software (Altschul et al. 1997). In the multilocation analysis, the main effects of female, male gene and their interaction were considered as fixed effects and the main effects of nest and location and their interaction, the interaction between male gene and nest, the interaction between nest and location, and the three‐way interaction among locations, male gene and nests were considered as random effects. Blocking factors such as replicates within each location, replicate interaction with nest within each location and field spatial variation within each location were considered as random effects. Autoregressive correlation as AR1 × AR1 within each location was included to reduce noise caused by spatial variation in the field. BLUE differences across and within nest were calculated and the *p* value for each of these contrasts was calculated and included in the data tables. The significance test was performed using a *p*‐value of 0.05 in a two‐tailed test.

## Author Contributions

B.L., A.L., K.F., V.L., G.T., B.W, J.Q., cloned the NLB18‐R gene. H.G., B.L., G.T., R.B.M., L.P., N.K., designed edits. M.R., N.S.‐D., M.Y., M.S., H.G., W.‐N.H., M.B. conducted transformation and molecular analysis experiments. G.T., B.B., B.N., H.M. designed and conducted greenhouse and field experiments and analysed data. H.G., G.T., B.L. wrote the manuscript. J.E.H. reviewed and edited the manuscript.

## Conflicts of Interest

The authors declare no conflicts of interest.

## Supporting information


**Figure S1:** Protein sequence alignment of NLB18‐PH26N (NLB18‐R), Htn1, Ht2 and the B73 susceptible allele.

**Figure S2:** Target sites at NLB18 locus and TS3 site formation after deletion of NLB18 susceptible allele.

**Figure S3:** Schematic illustration of progeny genotypes of the NLB18‐S deletion line (NLB18‐S‐del) and NLB18‐R allele swap.

**Figure S4:** Insert resistant cisgenes into chromosome 1.

**Figure S5:** Example of SbS analysis of edited plants.

**Figure S6:** Grain moisture of hybrids with NLB18‐R and HT1‐R on Chr01 sites.

**Figure S7:** CRISPR component configurations across three editing approaches.

**Table S1:** Markers for NLB18‐PH26N cloning.

**Table S2:** Greenhouse evaluation of NLB17 transgenic plants.

**Table S3:** PCR primers and probes for detecting edit variants.

**Table S4:** PCR primers and probes for detecting helper genes.

**Table S5:** Primers used for quantifying resistance gene expressions and recombinant screening.

## Data Availability

All data supporting the findings of this study are available in the article and its Supporting Information files. Plasmid accession numbers are MN294713 (Cas9), MN294716 (NPTII), MN294717 (Bbm), MN294718 (Wus2), ON685201 (PH4GP‐Ht1 CDS), ON685202 (PH4GP‐Ht1 genomic sequence), PX598904 (PH26N‐NLB18 genomic sequence), PX598905 (PH26N‐NLB17 genomic sequence), PX533534 (PH26N‐NLB18 CDS), PX533535 (PH26N‐NLB17 CDS).
