# Supplementary material for: Genome‐Edited Maize Expressing Two Native Genes Confers Broad‐Spectrum Resistance to Northern Corn Leaf Blight
Source: Mol Plant Pathol. 2026 Feb 11;27(2):e70205. doi: 10.1111/mpp.70205 (PMC12894063; doi:10.1111/mpp.70205)
Supplement: Supplementary file 1 — Figure S1: Protein sequence alignment of NLB18‐PH26N (NLB18‐R), Htn1, Ht2 and the B73 susceptible allele. [file MPP-27-e70205-s001.pdf]

```
NLB18-B73      MAAHLPRLPVLLLVLLAAHVVSTSTRAE--------------PLLPSTYNVSMCSESFWCGGVEIRYPFYLANATADYSGSYYSCGYTDLSVSCELEVEG   86
NLB18-Ph26N    MAAHLPRLPVLLLVLLAAHVVSTSAHAEPPLPSPYSTSAHGEPPLPSTYNVSMCSESFWCGGVEIRYPFYLANATADYSGSYYSCGYTDLSVSCKLEVEG  100
Ht2            MAAHLPRLPVLLLVLLAAHVVSTSAHAEPPLPSPYSTSAHGEPPLPSTYNVSMCSESFWCGGVEIRYPFYLANATADYSGSYYSCGYTDLSVSCKLEVEG  100
Htn1           MAAHQPHLSVLLLVLLAAHVVSTSAHGEPPLPSPYNTSAHGEPPLPSTYNASMCS-SFWCGGVEIRYPFYLANAIADYSGSYYSCGYTDLSVSCELEVEG   99

NLB18-B73      PPTTWTPTIRLGGDNYTVKNILY--DYHTISLADSDVLGGGECPVVRHNVSFDETWLH--NASAFNNLTFFFGCHWGPRDTLPEFAGYNISCDGFSTPTI  182
NLB18-PH26N    PTTTWTPTIRLGGDNYTVKNILY--DYHTISLADSDVLGGGECPVVHHNVSFDETWLH--NPSAFDNLTFFFGCHWGPRDTLPEFAGNNISCAGFSTPAI  196
Ht2            PTTTWTPTIRLGGDNYTVKNILY--DYHTISLADSDVLGGGECPVVHHNVSFDETWLH--NPSAFDNLTFFFGCHWGPRDTLPEFAGNNISCAGFSTPAI  196
Htn1           SPTTWTPTIRLGGGDYTVKNISYLYDQQTISLADRDVLGGGCPVVRHNVSFDETWLHLHNASAFDNLTFFFGCHWGPRNTPPEFADYNISCAGFNTPTI  199

NLB18-B73      SGGGSFVFKTEDLDEQEEQDFASHCDEVFSVPVGSEALRAT-DTFSLSRYGYGELLRQGFELEWNRTSEDQCGQCEGSSSGGRCAYNQKREFLGCLCSGG  281
NLB18-PH26N    SGGGSFVFKPEDLDEHAEQELASHCDEVFSVPVRSEALQQAIVSNLSLGDGYGELLRQGIELEWKRTSEDQCGQCEESGSGGRCAYSQKREFLGCLCSGG  296
Ht2            SGGGSFVFKPEDLDEHAEQELASHCDEVFSVPVRSEALQQAIVSNLSLGDGYGELLRQGIELEWKRTSEDQCGQCEESGSGGRCAYSQKREFLGCFCSGG  296
Htn1           SGGRSFVFKTGDLDEQEEQELALHCDEVFSVPVRRDALQAIVSNFSLTRDGYGEVLRQGFELEWNRTSEDQCGRCEGSGSGGWCAYSQKREFLGCLCSGG  299

NLB18-B73      KAGNPFCKPSRSK--RNEGPIVGAVVAVAFLCLVILTCFLACRHGSLPFKSKNKPGTRIESFLQKNESSIHPKRYTYADVKRMTKSFAVKLGQGGFGAVY  379
NLB18-BC26N    KAGNPFCKPSRSK--RKEASIVGA-VAVAFLCLVILTCFLACRHGSLPFKSENKPGTRIESFLQKNE-SIHPKRYTYTDVKRMTKSFAVKLGQGGFGAVY  392
Ht2            KAGNPFCKPSRSTKRREAASIVGA-VAVAFLCLVILTCFLACRHCSLPFKSKNKPGTRIESFLQKNESSIHPKRYTYADVKRMTKSFAVKLGQGGFGAVY  395
Htn1           KVGSPFCKPSRSK--RKEGPIVGA-VAVAFLCLVILTCFLACRHGSLPFKSENKPGTRIESFLQKNE-SIHPKRYTYADVKRMTKSFAVKLGQGGFGAVY  395

NLB18-B73      KGSLH-GRQVAVKMLKDTQGDGEEFMNEVASISRTSHVNVVTLLGFCLQGSKRALIYEYMPNGSLERYAFTGDMNSENLLSWERLFDIAIGTARGLEYLH  478
NLB18-PH26N    KGSLHDGRQVAVKMLKDTQGDGEEFMNEVASISRTSHVNVVTLLGFCLQGSKRALIYEYMPNGSLERYAFTGDMNSENLLTWERLFDIAIGTARGLEYLH  492
Ht2            KGSLHDGRQVAVKMLKDTQGDGEEFMNEVASISRTSHVNVVTLLGFCLQGSKRALIYEYMPNGSLERYAFTGDMNSENLLTWERLFDIAIGTARGLEYLH  495
Htn1           KGSLHDGRQVAVKMLKDTQGDGEEFMNEVASISRTSHVNVVTLLGFCLQGSKRALIYEYMPNGSLERYAFTGDMNSENLLTWERLFDIAIGTARGLEYLH  495

NLB18-B73      RGCNTRIVHFDIKPHNILLDQDFCPKISDFGLAKLCLNKESAISIVGARGTIGYIAPEVYSKQFGTISSKSDVYSYGMMVLEMVGARDRNTS--ADSDHS  576
NLB18-PH26N    RGCNTRIVHFDIKPHNILLDQDFCPKISDFGLAKLCLNKESAISIVGARGTIGYIAPEVYSKQFGTISSKSDVYSYGMMVLEMVGARERNTSASADSDHS  592
Ht2            RGCNTRIVHFDIKPHNILLDQDFCPKISDFGLAKLCLNKESAISIAGARGTIGYIAPEVYSKQFGTISSKSDVYSYGMMVLEMVGARDRNTS--ADSDHS  593
Htn1           RGCNTRIVHFDIKPHNILLDQDFCPKISDFGLAKLCLNKESAISIAGARGTIGYIAPEVYSKQFGIISSKSDVYSYGMMVLEMVGARDRNTS--ADSDHS  593

NLB18-B73      SQYFPQWLYEHLDDYCVGASEINGETTELVRKMIVVGLWCIQVIPTDRPTMTRVVEMLEGSTSNLELPPRVLLS*                            651
NLB18-PH26N    SQYFPQWIYEHLDDYCVGASEINGETTELVRKMIVVGLWCIQVIPTDRPTMTRVVEMLEGSTSNLELPPRVLLS*                            667
Ht2            SQYFPQWLYEHLDDYCVGASEINGETTELVRKMIVVGLWCIQVIPTDRPTMTRVVEMLEGSTSNLELPPRVLLS*                            667
Htn1           SQYFPQWLYEHLDDYCVGASEINGETTELVRKMIVVGLWCIQVIPTDRPTMTRVVEMLEGSTSNLELPPRVLLS*                            667
```

Supplementary Figure 1. Protein sequence alignment of NLB18-PH26N (NLB18-R), Htn1, Ht2 and the B73 susceptible allele.
