# Supplementary material for: Genome‐Edited Maize Expressing Two Native Genes Confers Broad‐Spectrum Resistance to Northern Corn Leaf Blight
Source: Mol Plant Pathol. 2026 Feb 11;27(2):e70205. doi: 10.1111/mpp.70205 (PMC12894063; doi:10.1111/mpp.70205)
Supplement: Supplementary file 3 — Figure S3: Schematic illustration of progeny genotypes of the NLB18‐S deletion line (NLB18‐S‐del) and NLB18‐R allele swap. [file MPP-27-e70205-s010.pdf]

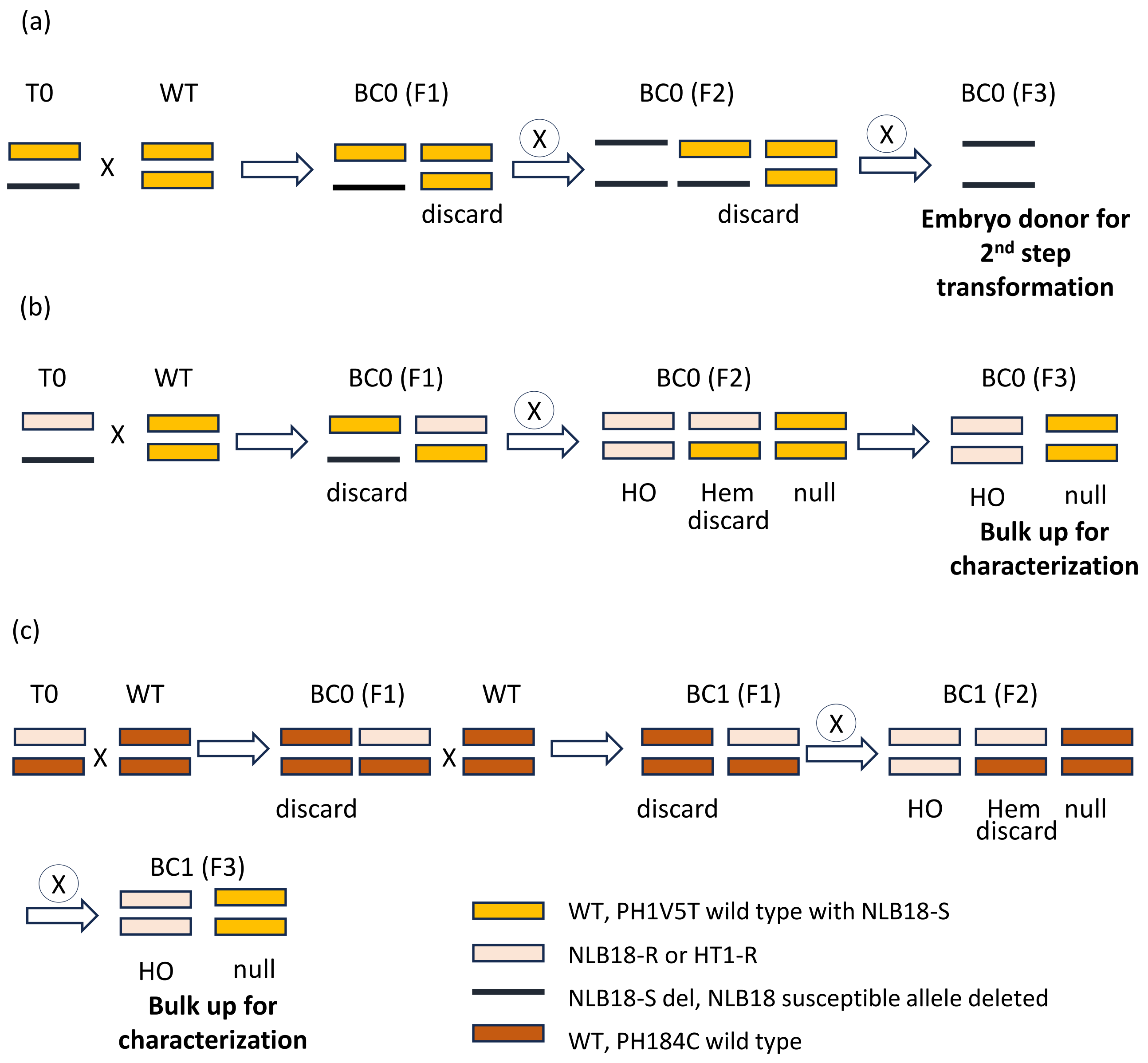

Supplementary Figure 3. Schematic illustration of progeny generation and genotypes.

(a) NLB18-S-del embryo donor production. (b) Generation of NLB18-R progeny from 2 step allele swap. (c) Generation of NLB18-R or HT1-R progenies from insertion to chromosome 1 target sites. HO, homozygous; Hem, hemizygous; null, null segregant of edit event; WT, wild type.
