# Supplementary material for: Genome‐Edited Maize Expressing Two Native Genes Confers Broad‐Spectrum Resistance to Northern Corn Leaf Blight
Source: Mol Plant Pathol. 2026 Feb 11;27(2):e70205. doi: 10.1111/mpp.70205 (PMC12894063; doi:10.1111/mpp.70205)
Supplement: Supplementary file 4 — Figure S4: Insert resistant cisgenes into chromosome 1. [file MPP-27-e70205-s007.pdf]

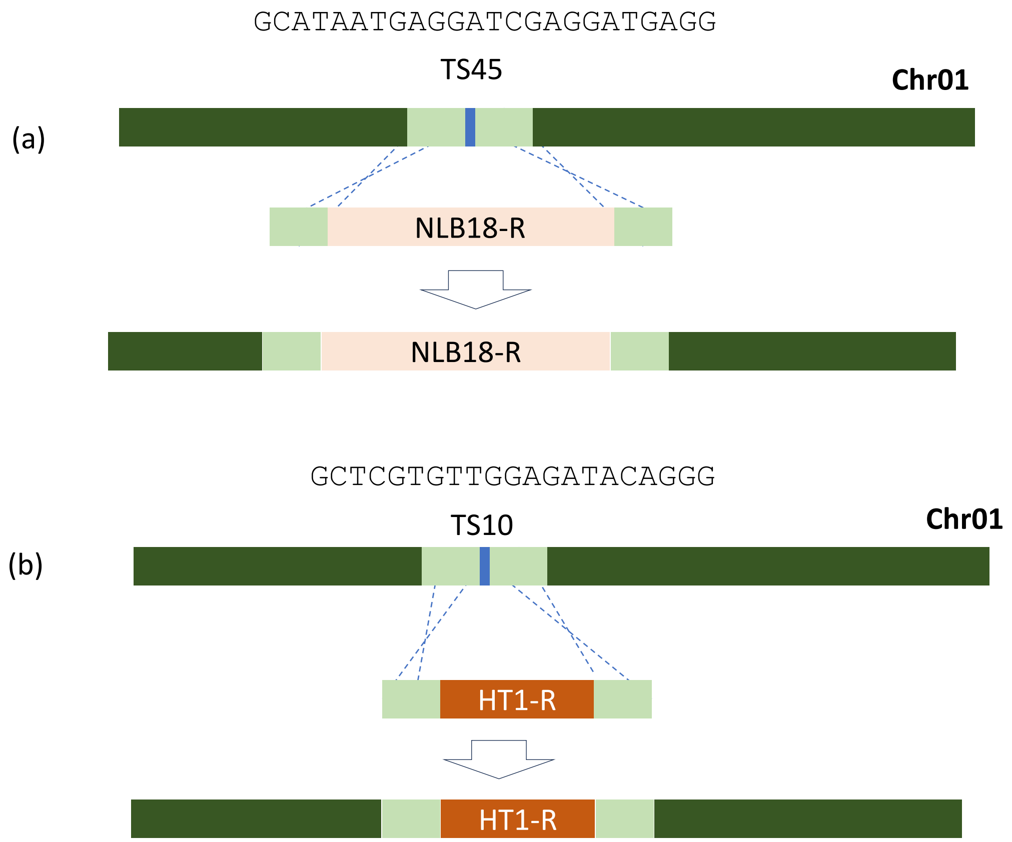

Supplementary Figure 4. Insert of NCLB resistance cisgenes into chromosome 1.

(a) Schematic drawing of inserting NLB18 resistant allele PH26N (NLB18-R) to target site 45 (TS45) via homology directed repair (HDR). The NLB18-R donor was flanked by homology sequences (pale green). (b) Schematic drawing of inserting HT1 resistant allele PH4GP (HT1-R) to target site 10 (TS10) via HDR. The HT1-R donor was flanked by homology sequences (pale green). TS45 and TS10 sequences are shown on top of each site.
