# Supplementary material for: Genome‐Edited Maize Expressing Two Native Genes Confers Broad‐Spectrum Resistance to Northern Corn Leaf Blight
Source: Mol Plant Pathol. 2026 Feb 11;27(2):e70205. doi: 10.1111/mpp.70205 (PMC12894063; doi:10.1111/mpp.70205)
Supplement: Supplementary file 5 — Figure S5: Example of SbS analysis of edited plants. [file MPP-27-e70205-s005.pdf]

(a)

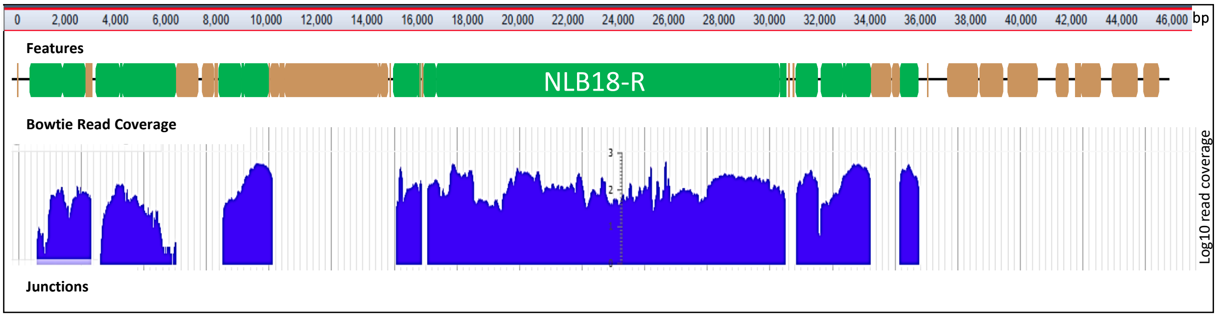

(b)

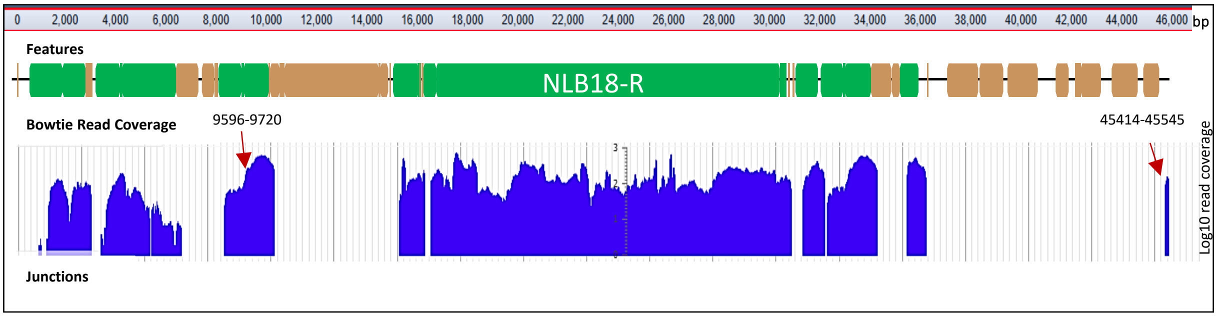

Supplementary Figure 5. Example of SbS analysis of edited plants.

Schematic and sequence coverage illustration for NLB18-R allele swap BC0 plants. (a) Plant confirmed of perfect allele swap and absence of unintended DNA in the genome from transformation. (b) Plant confirmed of perfect allele swap but fragments of DNA from transformation present. A 125bp and 132bp sequence from vector were detected in the genome (red arrows and numbers). Green and tan bars on top represent features from maize and other sources in the transformation vector, respectively . Maize endogenous genetic elements in transformation showed up in sequence reads along with expected allele swap at the target site. PH1V5T wild type DNA was referenced for sequence analysis.
