# Supplementary material for: Genome‐Edited Maize Expressing Two Native Genes Confers Broad‐Spectrum Resistance to Northern Corn Leaf Blight
Source: Mol Plant Pathol. 2026 Feb 11;27(2):e70205. doi: 10.1111/mpp.70205 (PMC12894063; doi:10.1111/mpp.70205)
Supplement: Supplementary file 6 — Figure S6: Grain moisture of hybrids with NLB18‐R and HT1‐R on Chr01 sites. [file MPP-27-e70205-s012.pdf]

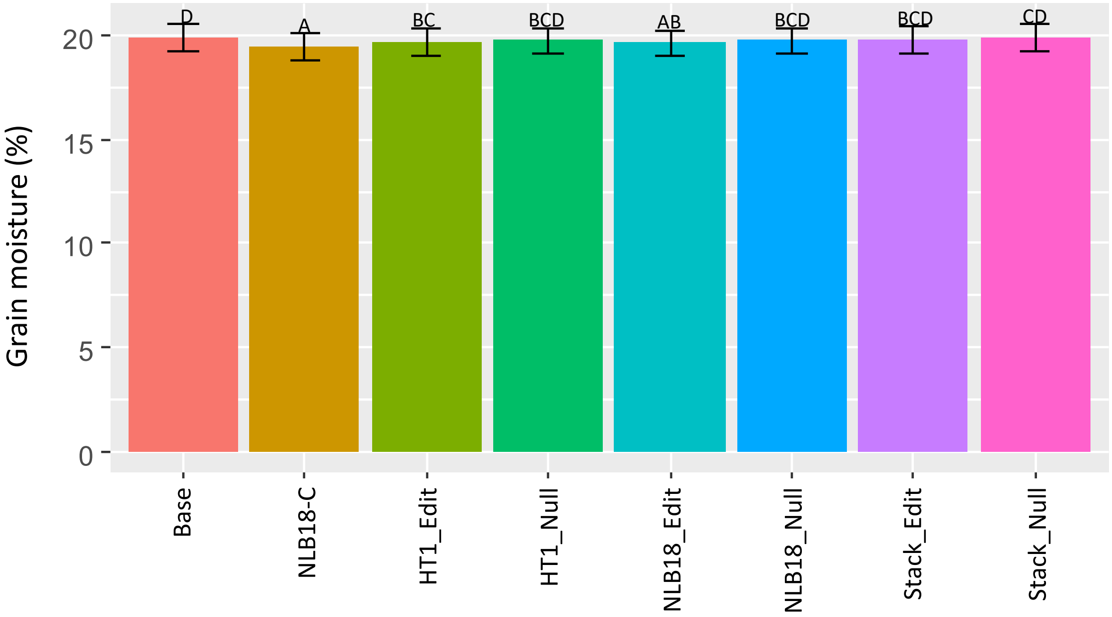

Supplementary Figure 6. Grain moisture of hybrids with NLB18-R and HT1-R on Chr01 sites.

Grain moistures were collected from multiple selected locations at harvest time in hybrid yield trials. Analyses were conducted using ASReml and the values are presented as Best Linear Unbiased Estimates (BLUEs). Significant difference among the tested hybrids are indicated by the letter on top of each bar. ($p<0.05$, Tukey's HSD test). Base, PH184C wild type crossed with testers; NLB18-C, NLB18-R conversion; HT1_Edit, HT1-R at TS10; NLB18_Edite, NLB18-R at TS45; Stack _Edit, NLB18-R and HT1-R stacked by crossing; Null, null segregant of each edited hybrids.
