# Supplementary material for: Genome‐Edited Maize Expressing Two Native Genes Confers Broad‐Spectrum Resistance to Northern Corn Leaf Blight
Source: Mol Plant Pathol. 2026 Feb 11;27(2):e70205. doi: 10.1111/mpp.70205 (PMC12894063; doi:10.1111/mpp.70205)
Supplement: Supplementary file 7 — Figure S7: CRISPR component configurations across three editing approaches. [file MPP-27-e70205-s006.pdf]

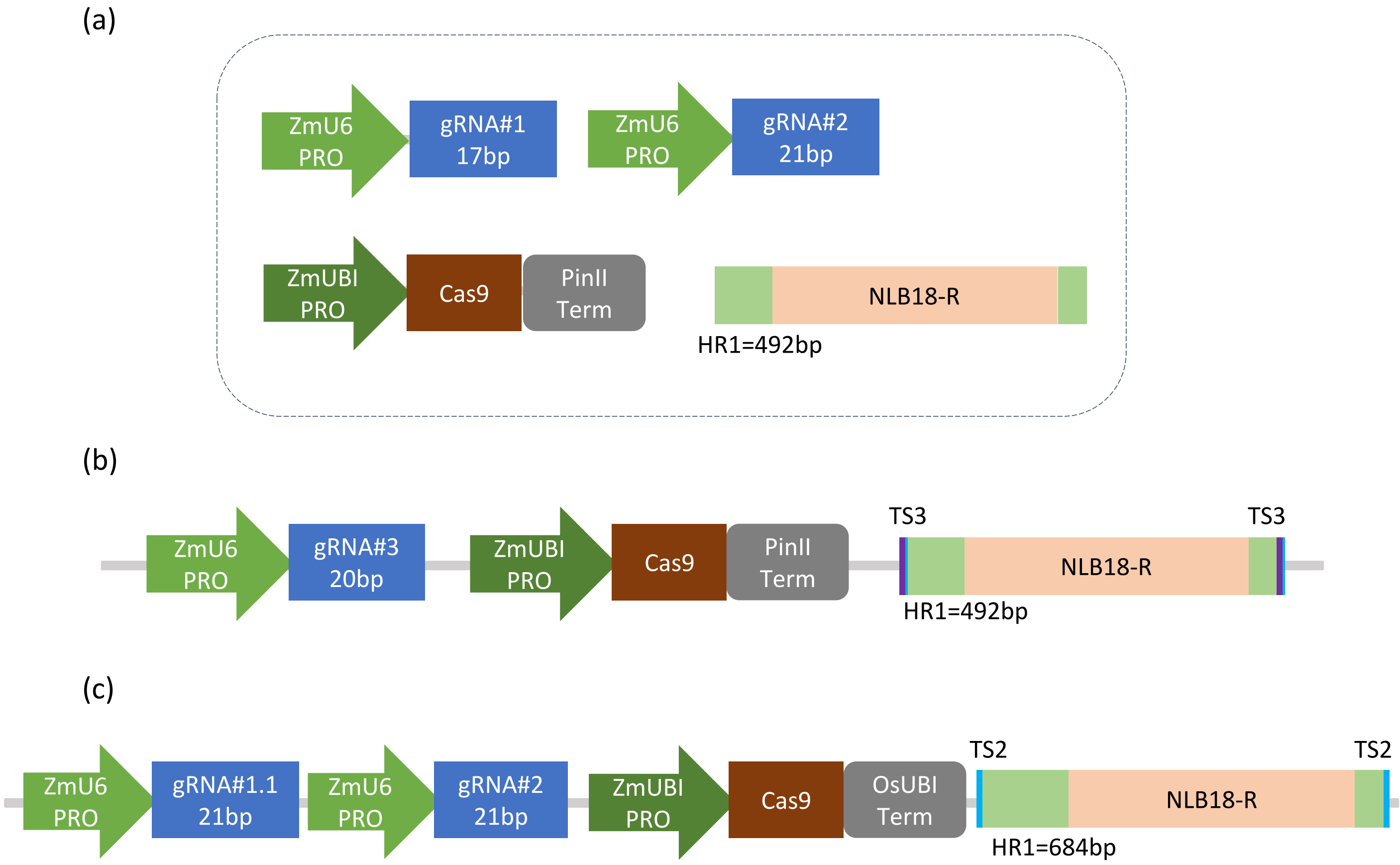

Supplementary Figure 7. CRISPR component configurations across three editing approaches.

(a) Editing vectors for initial allele swap delivered as plasmid DNA via particle bombardment. (b) Editing cassettes in *Agrobacterium* vector for the two-step approach. (c) Editing cassettes in *Agrobacterium* vector for direct allele swap.
