# Supplementary material for: Genome‐Edited Maize Expressing Two Native Genes Confers Broad‐Spectrum Resistance to Northern Corn Leaf Blight
Source: Mol Plant Pathol. 2026 Feb 11;27(2):e70205. doi: 10.1111/mpp.70205 (PMC12894063; doi:10.1111/mpp.70205)
Supplement: Supplementary file 8 — Table S1: Markers for NLB18‐PH26N cloning. [file MPP-27-e70205-s004.pdf]

Supplementary Table 1. Markers for NLB18-PH26N cloning

Marker name	Marke type	orientation	sequence
PHM1339-27	SNP-Invader	forward	tccggcgggt ccgcggtg
PHM13395-27	SNP-Invader	reverse	agacacggtt acttctaacg aa
PHM4677-11F	SNP-Invader	forward	agaagaacta gagcaacagc a
PHM4677-11 R	SNP-Invader	reverse	aatctcaact caacctccat aa
108f16_2_F	CAPS	forward	gagtgggtgt cgtagttcag c
108f16_2_R	CAPS	reverse	tcgactacaa gacgcgtacc
Pco642297_3_F	CAPS	forward	cgaccgacga tagatggtgt c
Pco642297_3_R	CAPS	reverse	atgttgctcc aacagatact ttgc
