# Supplementary material for: Genome‐Edited Maize Expressing Two Native Genes Confers Broad‐Spectrum Resistance to Northern Corn Leaf Blight
Source: Mol Plant Pathol. 2026 Feb 11;27(2):e70205. doi: 10.1111/mpp.70205 (PMC12894063; doi:10.1111/mpp.70205)
Supplement: Supplementary file 9 — Table S2: Greenhouse evaluation of NLB17 transgenic plants. [file MPP-27-e70205-s011.pdf]

Supplementary Table 2. Greenhouse evaluation of NLB17 transgenic plants

T1 hemizygous plants and null segregants were inoculated with race 0 of *S. turcica.* Present or absent of necrotic lesions on leaves 14 days after inoculation was categorized as susceptive or resistance, respectively.

Plant genotype	Number of resistant plant	Number of susceptible plant
NLB17-PH26N pos	0	8
NLB17-PH26N null	0	8
