# Supplementary material for: Genome‐Edited Maize Expressing Two Native Genes Confers Broad‐Spectrum Resistance to Northern Corn Leaf Blight
Source: Mol Plant Pathol. 2026 Feb 11;27(2):e70205. doi: 10.1111/mpp.70205 (PMC12894063; doi:10.1111/mpp.70205)
Supplement: Supplementary file 10 — Table S3: PCR primers and probes for detecting edit variants. [file MPP-27-e70205-s008.pdf]

Supplementary Table 3. PCR primers and probes for detecting edit variants

Edit type	Primer name	Primer orientation	Primer sequence
NLB18-S allele deletion	NLB18S-fp	forward	gtggatgggcaagatgctagag
	NLB18S-rp	reverse	ATATTGTTTTTGTTGTGATTTACGACC
NLB18 allele swap-2 steps			
HR1-1st PCR	18s-hdr1-fp	forward	GCAAAAGGGCTATAAACTACATGAAAAG
	26n-rp	reverse	TACACTACATCAAAAGTGCTCGAAGA
HR1-qPCR	26n-p-fp	forward	GCCTCGAACTCCAACATCACA
	26n-p-rp	reverse	ACCCACTCAGTTCCATGACAGA
	26n-p-probe		ctctacaactgcatgaag
HR2-1st PCR	26n-t-fp	forward	AACGAGCCGAGTCGAGTCAA
	18s-hdr2-rp	reverse	agcttacacttggttagatgaactaataagt
HR2-qPCR	26n-t-fp	forward	AACGAGCCGAGTCGAGTCAA
	26n-t-rp	reverse	ACTCGTGGATTGTGAGCTTGCT
	26n-t-probe		tcgactcatcgtgagct
NLB18-PH26N insert to TS45			
HR1-1st PCR	45-hdr1-fp	forward	GCGTGCGTGCTTACATGATG
	26n-p-rp	reverse	ACCCACTCAGTTCCATGACAGA
HR1-qPCR	26n-p-fp	forward	GCCTCGAACTCCAACATCACA
	26n-p-rp	reverse	ACCCACTCAGTTCCATGACAGA
	26n-p-probe		ctctacaactgcatgaag
HR2-1st PCR	26n-t-fp	forward	AACGAGCCGAGTCGAGTCAA
	45-hdr2-rp	reverse	AGTCGACATTAAACAATGTTAGTTGTAGCC
HR2-qPCR	26n-t-fp	forward	ACTCGTGGATTGTGAGCTTGCT
	26n-t-rp	reverse	ACTCGTGGATTGTGAGCTTGCT
	26n-t-probe		tcgactcatcgtgagct
HT1-PH4GP insert to TS10			
HR1-PCR	10-hdr1-fp	forward	TGGCTTGTCTATGCGCATCTC
	4GP-p-rp	reverse	TGACAGTTGATAAAGACCGGATGA
HR2-PCR	10-hdr2-rp	forward	GGACATCTATACTTGAGGGCTTCAC
	4GP-t-fp	reverse	TGAACCGGCTAGGAAAGTTTAGTT
