# Supplementary material for: Genome‐Edited Maize Expressing Two Native Genes Confers Broad‐Spectrum Resistance to Northern Corn Leaf Blight
Source: Mol Plant Pathol. 2026 Feb 11;27(2):e70205. doi: 10.1111/mpp.70205 (PMC12894063; doi:10.1111/mpp.70205)
Supplement: Supplementary file 11 — Table S4: PCR primers and probes for detecting helper genes. [file MPP-27-e70205-s003.pdf]

Supplementary Table 4. PCR primers and probes for detecting helper genes

PCR for	Primer name	Primer orientation	Primer sequence
Cas9	Cas9-FP	forward	CAGAATGAAAAGCTCTACCTCTACTACCT
	Cas9-RP	reverse	TGGTCGACGTCGTAGTCCGA
	Cas9-probe		FAM-TCCTGGTCCACGTACAT
gRNA	gRNA-FP	forward	CTAATCACAAGAGTGGAGCGTACCTT
	gRNA-RP	reverse	AGCCTTATTTTAACTTGCTATTTCTAGCTCT
	gRNA-probe		FAM-CCGAGCCGCAAGCA
BbM	ODP2-FP	forward	CGGCGATGTCTGCTTCAA
	ODP2-RP	reverse	AAGCTCTGATCCCCTCATGCT
	ODP2-probe		FAM-ATCCCCCAAGATTG
Wus2	Wus-FP	forward	CTCTGTCCGGTGTCAGTAGCAAT
	Wus-RP	reverse	TGCCTCCTCCCGCTCC
	Wus-probe		FAM-ACCGCCGCCCGCA
NPTII	npt2-FP	forward	CGACCACCAAGCGAAACAT
	npt2-RP	reverse	CGACAAGACCGGCTTCCAT
	npt2-probe		CATCGAGCGAGCACGT
ADH reference	ADH-FP	forward	CAAGTCGCGGTTTTCAATCA
	ADH-RP	reverse	GAAGGTGGAAGTCCCAACAA
	ADH-probe		IC-TGGGAAGCCTATCTACCAC
