# Supplementary material for: Genome‐Edited Maize Expressing Two Native Genes Confers Broad‐Spectrum Resistance to Northern Corn Leaf Blight
Source: Mol Plant Pathol. 2026 Feb 11;27(2):e70205. doi: 10.1111/mpp.70205 (PMC12894063; doi:10.1111/mpp.70205)
Supplement: Supplementary file 12 — Table S5: Primers used for quantifying resistance gene expressions and recombinant screening. [file MPP-27-e70205-s009.pdf]

Supplementary Table 5. Primers used for quantifying resistance gene expression and recombinant screening

PCR for	Primer name	Primer orientation	Primer sequence
NLB18-PH26N	NLB18-F1	forward	TCAAATGTTGCTTCCAGGTCACA
	NLB18-R1	reverse	GTCATACAGATACAGTGTATGAGGTAAATA
HT1-PH4GP	HT1-F3	forward	GTGATCTGAGGATCGACGAA
	HT1-R3	reverse	TTTGAGCCGGTAGCACGACA
eIF4G reference	eIF4g-F1	forward	AACACTTAGGCCAGCATTCG
	eIF4g-R1	reverse	TGTCAAATGGCTCGAGGAG
