# Supplementary material for: Genome‐Edited Maize Expressing Two Native Genes Confers Broad‐Spectrum Resistance to Northern Corn Leaf Blight
Source: Mol Plant Pathol. 2026 Feb 11;27(2):e70205. doi: 10.1111/mpp.70205 (PMC12894063; doi:10.1111/mpp.70205)
Supplement: Supplementary file 2 — Figure S2: Target sites at NLB18 locus and TS3 site formation after deletion of NLB18 susceptible allele. [file MPP-27-e70205-s002.pdf]

TS1 GTTAACGATGTCAACGTCGG
gRNA#1 GTTAACGATGTCAACGT
TS1.1 CTCGTTAACGATGTCAACGTCGG
gRNA#1.1 GTCGTTAACGATGTCAACGT

TS2 GTTCGCATTAGTCTAGGATTAGGG
gRNA#2 GTTCGCATTAGTCTAGGATTA

TS1 **~14kbp** **TS2**

CAAGGCGCATATCATACTAGTCTCCGACGTTGACATCGTTAACGAGTAAGCGGT....................GTAACTTTGGTTCGCATTAGTCTAGGATTAGGGTTGAATATTGATCAA

↓

CAAGGCGCATATCATACTAGTCTCCGACGTTAGGGTTGAATATTGATCAA

TS3 CATACTAGTCTCCGACGTTAGGG

gRNA#3 GATACTAGTCTCCGACGTTA

Supplementary Figure 2. Target sites at NLB18 locus and TS3 site formation after deletion of NLB18 susceptible allele.

Deletion of the NLB18 susceptible allele was achieved by cutting at TS1 and TS2 sites. Cleavage sites are underlined. PAM sites are shown in rectangles. PAM for TS1 and TS2 sites remained intact after deletion. End joining formed a new target site, TS3, with a PAM from TS2. gRNA#3 was used in the second transformation to insert NLB18-R allele.
